# The Darlington and Northallerton Long Term Asthma Study: pulmonary function

**DOI:** 10.1186/1471-2466-5-2

**Published:** 2005-01-31

**Authors:** C Kevin Connolly, Robin J Prescott

**Affiliations:** 1 The Department of Medicine, The Memorial Hospital, Darlington, DL3 6HX, UK; 2 Medical Statistics Unit, University of Edinburgh, Edinburgh, EH8 9AG, UK

## Abstract

**Background:**

The Darlington and Northallerton Asthma Study is an observational cohort study started in 1983. At that time little was published about long term outcome in asthma and the contribution of change in reversible disease or airway remodelling to any excess deterioration in function. The study design included regular review of overall and fixed function lung. We report the trends over fifteen years.

**Methods:**

All asthmatics attending secondary care in 1983, 1988 and 1993 were recruited. Pulmonary function was recorded at attendance and potential best function estimated according to protocol. Rate of decline was calculated over each 5-year period and by linear regression analysis in those seen every time. The influence of potential explanatory variables on this decline was explored.

**Results:**

1724 satisfactory 5-year measurements were obtained in 912 subjects and in 200 subjects on all occasions. Overall rate of decline (ml/year (95%CI)) calculated from 5-year periods was FEV1 ♂41.0 (34.7–47.3), ♀28.9 (23.2–34.6) and best FVC ♂63.1 (55.1–71.2)ml/year, ♀45.8 (40.0–51.6).The principal association was with age. A dominant cubic factor suggested fluctuations in the rate of change in middle life with less rapid decline in youth and more rapid decline in the elderly. Rapid decline was possibly associated with short duration. Treatment step did not predict rate of deterioration.

**Conclusions:**

Function declined non-linearly and more rapidly than predicted from normal subjects. It reports for the first time a cubic relationship between age and pulmonary function. This should be taken into account when interpreting other articles reporting change in function over time.

## Background

It is recognised that the average decline in pulmonary function is greater in asthmatic subjects than in the general population [1,2]. This might be due to deterioration in potentially reversible disease [3] or the development of persistent obstruction following airway remodelling [4]. The published longitudinal studies do not differentiate between the two possible mechanisms. The Darlington and Northallerton Long Term Asthma Study was started in 1983 when little was known about long term outlook in asthmatics. Its objective was to observe mortality and decline in pulmonary function in asthmatics sufficiently severe to be referred for a hospital opinion. Decline in best achievable function is proposed as a measure of the airway remodelling. We accept that this decline might also be associated with Chronic Obstructive Pulmonary Disease (COPD). This label implies a physiological diagnosis, but the condition, like asthma, is better defined in terms of the underlying inflammation [5]. The diagnoses are therefore not necessarily mutually exclusive. Changes in best function are reported without prejudice to the underlying type of inflammation whether asthmatic, COPD, or both.

We wished this to be a population study as far as possible, so we invited all subjects satisfying a broad definition of asthma referred to a single-handed respiratory physician in a well defined geographical area to participate. Very few refused, but patients managed entirely in general practice were necessarily excluded. Best function, assessed according to a defined protocol [6] implicitly accepted in published guidelines [7], and potential explanatory variables were recorded prospectively at each visit. Smokers were not specifically excluded, but smoking habit was taken into account when the diagnosis was in doubt. Subjects with severe established fixed obstruction were excluded. Thus there was bias against the inclusion of asthmatic smokers destined to develop fixed obstruction rapidly, so we do not present any analyses confined to smokers. Although it was not until the mid 1980's that the use of prophylactic inhaled corticosteroids became standard practice, the majority of our subjects were maintained on inhaled steroids throughout the period, but the dose intensified [8]. Therefore only the change in dose could not account for any secular trends in decline in function. In this paper we explore the influence of demographic and other factors on change in function as observed over five year intervals.

## Methods

These, described in detail elsewhere [8-11] are summarised and where directly relevant expanded below.

### Subjects

All asthmatics currently attending secondary care clinics in the Darlington and Northallerton Health Districts were eligible for recruitment in 1983, 1988 and 1993, and reviewed in 1988, 1993 and 1998. Asthma was diagnosed clinically and confirmed by reversibility of FEV1 or peak flow of at least 15% on more than one occasion, either spontaneously, or in response to bronchodilator at any time since referral [9]. Subjects were only eligible for the study if they had been observed for at least one year before entry, and if not stable when first reviewed, entry was deferred by three months in an attempt to achieve stability. Socio-demographic variables were recorded as previously reported [10]. The first group of this dynamic cohort study was recruited during the calendar year 1983 but subsequent review and recruitment was between the 1^st ^April and the 31^st ^March in subsequent 12-month periods[11]. Allowance for the extra 3 months of the first interval has been made in calculating rate of change. Subjects are included in this report provided they had two technically satisfactory measurements of actual FEV1 or two estimates of best FEV1 or best FVC according to protocol.

### Social and demographic variables

The following variables recorded included: age, gender, height, duration of asthma, atopy, childhood asthma, smoking habit and lifetime amount smoked, social class. Duration of asthma was determined from the first onset or from relapse after a symptom free interval of at least five years, if this was applicable. Atopy was determined by at least one of the following skin tests resulting in a diameter at least 3 mm greater than control: D Pterynissimus, cat, grass pollen, A fumigatus. Childhood asthma was defined as a childhood history of recurrent lower respiratory tract symptoms with wheeze, in the absence of a localised structural damage such as bronchiectasis. It was sub-divided into those with (Gap Asthmatics) and without (Continuous since Childhood) a symptom free gap of at least five years. Never smokers were those smoking less than one cigarette a day for one year. Ex-smokers had been abstinent for at least three months at the time of examination. The lifetime amount smoked was determined from the average consumption (expressed in packs of 20 cigarettes) multiplied by the duration of smoking to give a figure in pack-years.

#### Therapeutic regimen

This was characterised by the use of long acting beta agonists and the corticosteroid step: none, low dose inhaled (<800 micrograms per day), intermediate dose inhaled (800–1000 micrograms per day), high dose inhaled (>1000 micrograms per day), oral 1–9 mgs per day, oral ≥ 10 mgs per day, unstable (treatment not mutually agreed as satisfactory over the last three months).

### Pulmonary function

#### Actual function

Actual function was that recorded at attendance.

### Best function

This was estimated according to the published protocol [6]. The notes were searched from January 1^st ^of the previous year, and the highest value, including the after-bronchodilator reading at attendance, was accepted as best subject to the following.

(a) If >80% predicted (Cotes [12]); after-bronchodilator

(b) If 70–80% predicted; after-bronchodilator and stable on mutually agreed satisfactory preventative treatment with twice daily recording of peak flow for one week

(c) If <70% of predicted; after-bronchodilator with formal trial of corticosteroids (prednisolone 30 mgs for at least five days with stability of peak flow for at least 48 hours)

If the above was not already satisfied, best function was immediately established according to the protocol.

### Measurement

Spirometry was performed using a rolling wedge spirometer (Vitalograph Limited) and peak flow with the mini peak flow meter (Clement Clark Limited). Actual function was measured opportunistically at the clinic in current attenders and at a special clinic for those who had been discharged. One of three research fellows, one of two research nurses or CKC were responsible for checking current pulmonary tests in the clinical records and performing further tests when required by protocol for best function.

### Ethical approval

This was obtained from the Darlington, Northallerton and South Durham Ethics Committees at various stages of the study.

### Statistical analysis

The principal independent variable was change during each 5-year period so each individual contributed up to 3 observations to the analysis. For the 200 subjects with observations on all four occasions a secondary analysis could be based on decline over 15 years, and for each of these subjects the mean rate of decline was estimated from the four observations using linear regression analysis.

Best and actual/best peak flow measured independently of the spirometric outcome variables were chosen as the functional potential predictors of outcome. This approach was taken to avoid the mathematical relationship between baseline level and change that applies if the spirometric variables are used as predictors of outcome. Many of these potential predictive variables are correlated with each other and there are arguments for and against models examining the effect of socio-economic variables with or without allowance for the respiratory function of the patients and their age. We present a relevant selection from the large range of unrestricted and hierarchical models that we constructed.

We allowed for the inevitable regression to the mean associated with outcome measures subject to appreciable variability. Initially the starting value of an outcome functional variable was regressed against a full set of potential predictors of this function in order to generate a set of residuals indicating the extent to which an individual starting measurement is higher or lower than would be expected. Then the change in function was regressed against the residual. The new set of residuals indicates the extent to which the apparent rate of change is affected by regression to the mean enabling an adjusted rate of change to be calculated. This adjusted change in function was then taken as the dependent variable in subsequent analyses. These included the univariate effect of potential risk factors on the adjusted change in function. In the multivariate analyses we omitted variables which were consistently non-significant, but all others are retained in the presentation. Models are shown with and without the inclusion of age. Univariate analysis showed that there were cubic relationships between change in function and age and duration. The cubic terms are retained in the multivariate presentation. We also examined interactions between predictor variables where there was some a priori reason for believing that such an interaction was plausible but none was demonstrated.

## Results

### General

Of 1138 subjects recruited, 155 died before the first review, 49 were lost to follow up and 22 did not have satisfactory spirometric tests. The remaining 912 had at least one paired result of actual or best FEV1 or best FVC and 200 had satisfactory assessments of best FVC on all four occasions. Demographic details at entry are given in Table 1. There were no relevant differences in social factors between the subjects observed on all four occasions and the rest. Current smokers represented approximately 13% of the population in 1983/88 and 10% in 1993/98 with a mean tobacco load of ♂29.2 (ex-smokers 27.6) and ♀21.4 (ex-smokers 13.2) pack years. Ever-smokers were significantly older than never-smokers (53.9 v 44.9 years p < 0.001), but allowing for age, the atopic status of ever-smokers and never-smokers was the same. The proportion of subjects stable on inhaled or oral steroids rose from 66.5% in 1983 to 82.1% in 1988 with no increase thereafter. Higher doses (> = 800 micrograms) of inhaled steroids increased from 30.8% in 1988 to 41.6% in 1998.

**Table 1:** Details of subjects at entry

		Male	Female
n		457	455
Age (years)(sd)		50.4(15.4)	49.3(16.3)
Duration of asthma (years)(sd)		17.0(16.2)	18.6(15.4)
Atopic n(%)		258(56.5)	227(49.9)
Asthma n(%)	childhood	124(27.1)	131(28.8)
	gap	53(11.6)	42(9.2)
	adult	280(61.3)	282(63.3)
Social Class n(%)	1–2	131(28.8)	139(30.5)
	3	168(36.9)	180(39.6)
	4–5	156(34.3)	136(29.9)
Smoking Habit n(%)	never	147(32.2)	247(54.3)
	ever	239(52.3)	161(35.4)
	current	71(15.5)	47(10.3)
Pulmonary Function(sd)	Actual FEV1 l.	2.50(1.09)	1.97(0.79)
	Best FEV1 l.	2.60(1.08)	2.08(0.82)
	Best FVC l.	4.17(1.25)	3.05(0.84)
	Best PEF l/min	465.7(113.7)	382.0(85.1)
	Actual/Best PEF %	84.4(13.7)	83.2(15.9)

### Change in function

**Figure 1:**
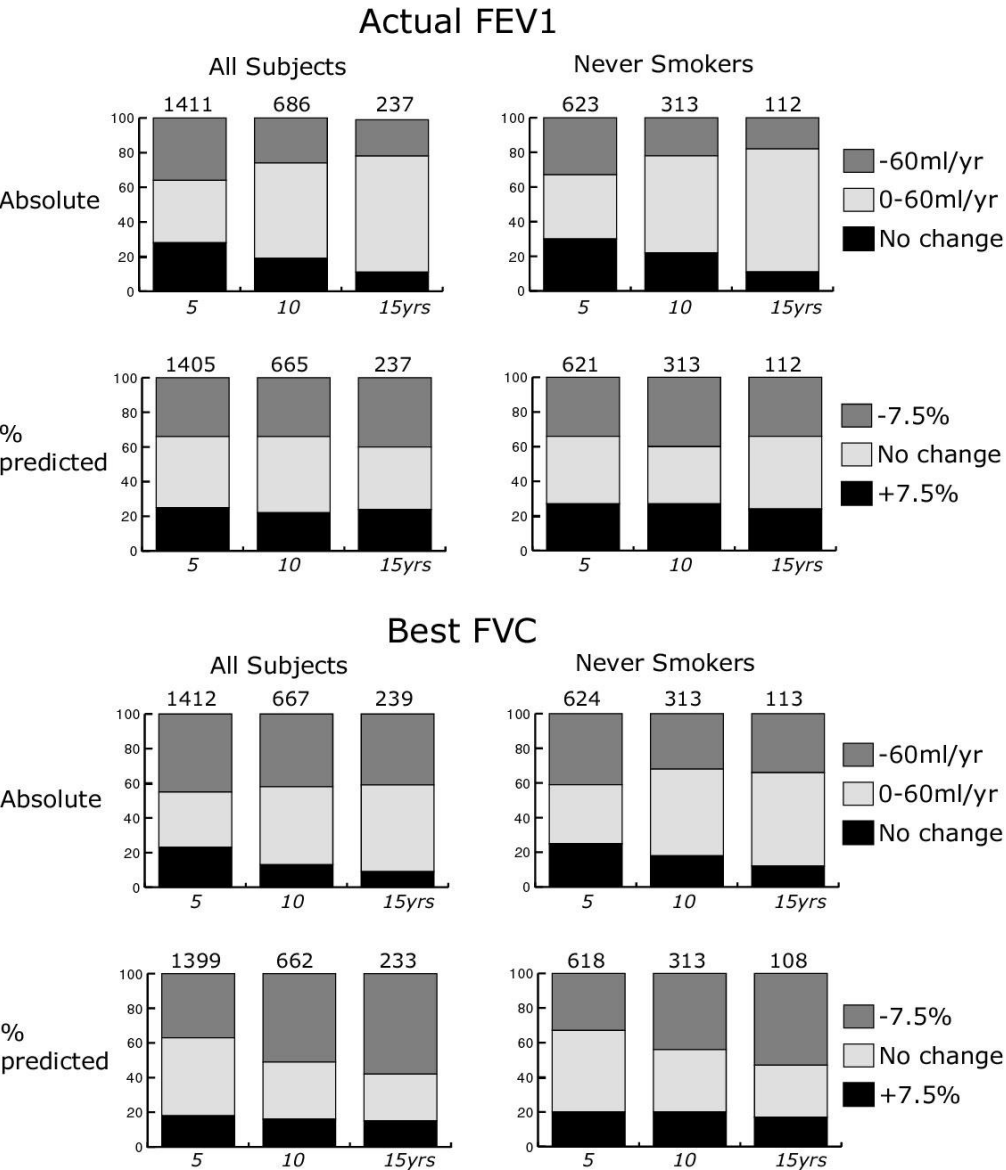
The proportion of all subjects and never-smokers showing decline (>7.5%), no change, or improvement (>7.5%) in function against predicted over 5, 10 and 15 years.

The mean annual decline in function calculated using all five-year intervals (Table 2) was greater than expected when compared with predicted values and when expressed as %predicted [12] there were no consistent differences in the rates of decline of male and female never-smokers. As the confidence intervals suggest, standard deviations were large indicating a wide distribution of changes in different individuals. There was no secular trend in outcome in successive calendar periods. Figure 1 shows changes over 5, 10 and 15 year periods after entry against predicted [12] (improved >7.5%, no change, deteriorated >7.5%). Actual FEV1 improved in approximately one quarter and best FVC in rather less than 20% of subjects. The proportion of subjects showing deterioration in FEV1 >7.5% (35%) did not increase with time after entry, but excess decline in FVC was observed in more subjects after 15 year's observation (58%) than after five (37%) (χ^2 ^for trend, 59.0 (p < 0.001)).

**Table 2:** Annual Decline in Pulmonary Function calculated from the mean of all 5-year paired observations, and by linear regression over 15 years in the 200 subjects with all four observations

	Annual Decline		n	Actual FEV1 l.	Best FEV1 l.	Best FVC l.
Males	**Over 5 Years**	**All Subjects**	776	41.0 (34.7–47.3)	42.9 (37.5–48.3)	63.1 (55.7–71.2)
		**Never smokers**	256	34.2 (22.2–46.2)	33.4 (22.7–44.1)	49.2 (35.6–62.8)
	**Over 15 Years**		85	46.7 (38.7–54.7)	-	64.7 (54.6–74.8)
Females	**Over 5 Years**	**All Subjects**	848	28.9 (23.2–34.6)	34.4 (29.8–39.0)	45.8 (40.0–51.6)
		**Never smokers**	375	28.7 (22.4–38.8)	30.6 (22.2–35.2)	45.2 (36.4–54.0)
	**Over 15 Years**		115	24.7 (14.8–31.2)	-	44.6 (37.9–51.3)

For the 200 subjects with complete observations over 15 years the mean rate of decline was similar to that observed over five year periods, but the standard deviation of the rates of decline was half that of the 5-year estimates, reflecting greater accuracy from multiple measurements. Even when calculating change this way several individuals improved or showed excessive loss in function against predicted [12].

### Associations with entry variables

As there was no secular trend in the change of function over the five year periods, the date of observation is not considered in the analyses below.

### All subjects: 5-year intervals

#### Univariate analysis

**Figure 2:**
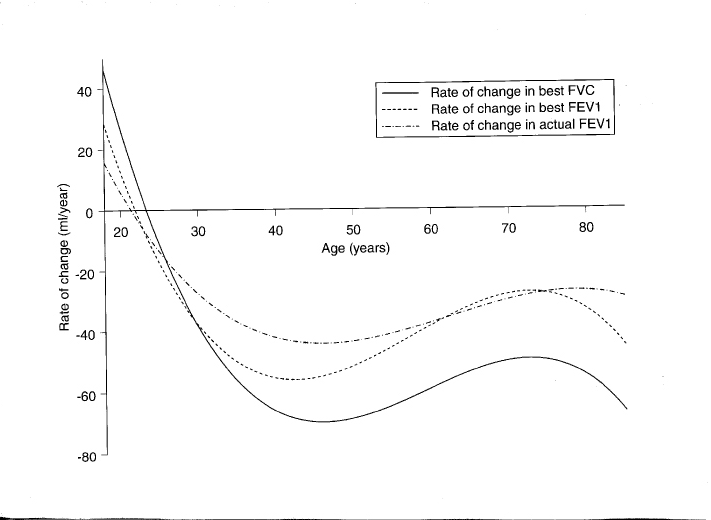
The relationship between age and annual change in function, observed over 5-year periods

The univariate relationships between the potential explanatory variables and change in function after allowance for regression to the mean are summarised in Table 3. These are derived from all available pairs of observations at five year intervals. The strong associations with age are not linear. This is demonstrated in fig 2 which shows the fitted plots of the cubic equations for the unadjusted rate of decline for all three measures. The maximum decline was in the mid 40's for all three variables (actual FEV1 44 ml/yr; best FEV1 56 ml /yr; best FVC 70 ml/yr). During the eighth decade rate of decline in function recovered towards the published predicted values [12] to 27, 29 and 49 ml/yr respectively.

**Table 3:** Rate of loss of Function Univariate Coefficients (ml per year) (after allowance for regression to the mean)

		Actual FEV1		Best FEV1		Best FVC	
		Estimate	95% CI	Estimate	95% CI	Estimate	95% CI

Gender (male v female)		7.7	0.8^+^,16.1	6.2	2.5^+^,14.9	**12.2**	**2.5,21.9**
Age at entry (per decade) (difference from age 50)	Linear	5.6^+^	11.4^+^,0.2	**10.1**^+^	**15.9**^+^,**4.8**^+^	2.1^+^	8.7^+^,4.5
	quadratic	**4.3**^+^	**6.0**^+^,**2.5**^+^	**5.0**^+^	**6.8**^+^,**3.3**^+^	**4.5**^+^	**6.5**^+^,**2.6**^+^
	cubic	**1.3**	**0.3,2.3**	**2.0**	**1.0,2.9**	**1.4**	**0.3,2.6**
Duration of asthma at entry (per decade) (difference from duration 20 yrs)	Linear	3.1^+^	7.4^+^,1.1	0.5	3.8^+^,4.8	0.1	4.8^+^,5.0
	quadratic	**3.6**	**0.6,6.6**	2.7	0.5^+^,5.8	**5.6**	**2.2,9.0**
	cubic	**0.9**^+^	**1.6**^+^,**0.1**^+^	**0.9**^+^	**1.7**^+^,**0.1**^+^	**1.4**^+^	**2.3**^+^,**0.6**^+^
Atopic		5.7^+^	14.2^+^,2.8	0.2	8.5^+^,8.9	3.0^+^	12.7^+^,6.7
Childhood asthma (versus no childhood asthma)	Gap	3.4^+^	16.7^+^,9.9	1.4	12.2^+^,15.0	4.0^+^	19.4^+^,11.3
	Yes	**20.8**^+^	**30.6**^+^,**10.9**^+^	**10.6**^+^	**20.9**^+^,**0.4**^+^	**22.0**^+^	**33.3**^+^,**10.6**^+^
Social Class (versus classes 1 and 2)	Three	6.3^+^	16.5^+^,3.8	**11.2**^+^	**21.7**^+^,**0.8**^+^	9.2^+^	20.8^+^,2.3
	Four and Five	10.2^+^	21.0^+^,0.7	**14.5**^+^	**25.6**^+^,**8.3**^+^	8.6^+^	21.0^+^,3.9
Amount smoked (per 10 pack years)		1.5	0.6^+^,3.7	0.2	2.1^+^,2.4	**2.9**	**0.4,5.4**
Best PEF(per 10% deficit)		**3.3**^+^	**5.3**^+^,**1.2**^+^	**2.8**^+^	**4.8**^+^,**0.7**^+^	0.4^+^	2.7^+^,2.0
Actual/Best PEF(10% deficit)		**12.7**^+^	**15.6**^+^,**9.8**^+^	2.8^+^	6.2^+^,0.6	0.6^+^	4.1^+^,2.8

(+) Indicates relative gain in function

There were no statistically significant relationships with atopy. Longer duration of asthma was significantly associated with favourable outcome for all variables. In all cases a cubic relationship between loss of function and duration suggests high initial rates of loss (actual FEV1 55 ml/yr; best FEV1 51 ml/yr; best FVC 74 ml/yr at one year), with improvement to a plateau at around 20 years duration (30 ml/yr, 37 ml/yr and 45 ml/yr). Childhood asthma was significantly associated with a relatively favourable outcome, though this benefit was only seen in those for whom asthma had been continuous. The outcome of the 'gap asthmatics' was similar to those with adult onset. Higher social class was associated with worse outcome, significantly so for best FEV1. Low initial function and worse control as assessed by actual/best PEF both predicted less loss in actual FEV1. Although there were no significant associations with current smoking status, there was significantly greater loss in best FVC with amount smoked.

#### Multivariate analysis

As childhood asthma, duration of asthma and age at entry are potentially confounded and were significant in some of the univariate analyses, we performed a series of multivariate analyses progressively including each in turn. Tables 4, 5, 6 show the results after the inclusion of duration and then age. Male gender was significantly associated with greater decline in best FEV1 and, in contrast to the univariate analysis, best FVC. After the inclusion of duration, childhood asthma was still associated with favourable outcome in both actual FEV1 and best FVC, but it was displaced by age in all the models. Duration remained significantly associated with less change in best FVC even after allowance for age. In both models, actual FEV1 declined less with lower entry actual/best PEF. Best FEV1 declined more rapidly in those with a high initial best PEF. When age was not included in the model, membership of social classes 1 and 2 remained associated with unfavourable outcome for best FEV1.

**Table 4:** Multivariate Coefficients (sd) for Rate of loss of Actual FEV1 (after allowance for regression to the mean)

		Age Excluded		Age Included	
		
		Coefficient	p	Coefficient	p
Gender (male v female)		5.5 (4.8)	0.25	5.7 (4.7)	0.23
Asthma since childhood		**13.5**^+ ^**(5.1)**	**0.008**	4.9^+ ^(5.8)	0.40
Social Classes 1 & 2 (v. classes 4 & 5)		4.8 (5.4)	0.65	1.8 (5.4)	0.88
Social Class 3 (v. classes 4 & 5)		1.1 (5.1)		0.6^+ ^(5.0)	
Amount smoked (per 10 pack years)		1.8 (1.2)	0.13	1.0 (1.2)	0.39
Best PEF(per 10% deficit)		1.2^+ ^(1.2)	0.33	0.9^+ ^(1.2)	0.41
Actual/Best PEF(10% deficit)		**12.0**^+ ^**(1.6)**	**<0.0001**	**13.2**^+ ^**(1.6)**	**<0.0001**
Age at entry (per decade) (difference from age 50)	Linear			3.5^+ ^(3.1)	
	quadratic			4.9^+ ^(0.9)	
	cubic			**1.2 (0.6)**	**0.02**
Duration of asthma at entry (per decade) (difference from duration 20 yrs)	Linear	1.3 (2.8)		2.0^+ ^(2.4)	
	quadratic	3.2 (1.5)		0.9 (0.5)	
	cubic	**0.85**^+ ^**(0.38)**	**0.03**	2.5^+ ^(0.39)	0.52

(+) Indicates relative gain in function

**Table 5:** Multivariate Coefficients for loss of Best FEV1 (after allowance for regression to the mean)

		Age Excluded		Age Included	
		
		Coefficient (sd)	p	Coefficient (sd)	p
Gender (male v female)		**11.3 (5.1)**	**0.03**	**11.9 (5.4)**	**0.02**
Asthma since childhood		5.1^+ ^(5.3)	0.34	1.1^+ ^(6.0)	0.86
Social Classes 1 & 2 (v. classes 4 & 5)		**13. (5.7)**	**0.05**	10.7 (5.6)	0.11
Social Class 3 (v. classes 4 & 5)		**2.9 (5.3)**		1.4 (5.2)	
Amount smoked (per 10 pack years)		0.7 (1.3)	0.60	0.5 (1.3)	0.71
Best PEF(per 10% deficit)		**3.4**^+ ^**(1.3)**	**0.008**	**3.1**^+ ^**(1,2)**	**0.01**
Actual/Best PEF(10% deficit)		0.7^+ ^(1.8)	0.71	1.4^+ ^(1.8)	0.44
Age at entry (per decade) (difference from age 50)	Linear			8.4^+^(3.2)	
	quadratic			5.1^+ ^(0.9)	
	cubic			**1.8 (0.5)**	**0.0003**
Duration of asthma at entry (per decade) (difference from duration 20 yrs)	Linear	3.1 (2.4)		0.2 (2.4)	
	quadratic	2.5 (1.6)		0.4 (1.6)	
	cubic	**0.91**^+ ^**(0.39)**	**0.02**	0.29^+ ^(0.40)	0.46

(+) Indicates relative gain in function

**Table 6:** Multivariate Coefficients for Rate of loss of best FVC (after allowance for regression to the mean)

		Age Excluded		Age Included	
		
		Coefficient (sd)	p	Coefficient (sd)	p
Gender (male v female)		**10.8 (5.6)**	**0.05**	**11.4 (5.6)**	**0.04**
Asthma since childhood		**16.0**^+ ^**(6.0)**	**0.007**	4.7^+ ^(6.9)	0.49
Social Classes 1 & 2 (v. classes 4 & 5)		10.7 (6.4)	0.13	7.5 (6.4)	0.29
Social Class 3 (v. classes 4 & 5)		0.2^+ ^(6.0)		1.4^+ ^(5.9)	
Amount smoked (per 10 pack years)		1.9 (1.4)	0.18	0.9 (1.4)	0.52
Best PEF(per 10% deficit)		0.6^+ ^(1,.4)	0.65	0.6^+ ^(1.9)	0.67
Actual/Best PEF(10% deficit)		1.3 (1.8)	0.47	0.1 (1.9)	0.94
Age at entry (per decade) (difference from age 50)	Linear			2.1^+ ^(3.7)	
	quadratic			4.0^+ ^(1.0)	
	cubic			**1.4 (0.6)**	**0.02**
Duration of asthma at entry (per decade) (difference from duration 20 yrs)	Linear	4.0 (2.8)		0.8 (2.8)	
	quadratic	5.2 (1.7)		3.2 (1.8)	
	cubic	**1.46**^+ ^**(0.44)**	**0.001**	**0.97**^+ ^**(0.46)**	**0.03**

(+) Indicates relative gain in function

Height (not tabulated) was consistently directly associated with decline at a significance level of the order of 15% in univariate and multivariate analyses. In the latter analyses, height suppressed the associations with gender and social class. There were no significant associations with instability of regimen, nor with steroid step or the regular prescription of bronchodilators. None of the models considered made any relevant difference to the shape or gradients of the curves shown in fig 2.

### 200 Subjects: decline estimated using all four observations

Generally similar univariate associations were demonstrated. However on multivariate analysis, amount smoked remained in the FVC model (coefficient 13.3 ml/year per 10 pack years P = 0.020), and the effect of gender was stronger in both models (actual FEV1 23.8, actual FVC 19.8 ml/year, (P < 0.001). Height was again consistently directly associated with decrease in function at a significance level of the order of 15%. When height was included to allow for its association with gender, the difference in rate of decline in FEV1 between males and females was substantially reduced to 12.5 ml per year (p = 0.010) and was no longer significant for best FVC (p = 0.14).

## Discussion

The hypothesis that persistent airway obstruction may develop in asthmatics implies that persistent airway obstruction and reversible obstruction associated with asthma are not mutually exclusive diagnoses However if COPD is regarded as a syndrome; this implies a non-asthmatic inflammatory process [5]. As the two types of inflammation need not be mutually exclusive, some asthmatics might have both types raising the possibility of an interaction between the two processes in some individuals, 'The Dutch Hypothesis' [13]. We have already shown that on cross sectional analysis green sputum, which is a feature of severity in COPD [14], is associated with diminished best function independent of smoking habit [15].

We are satisfied that our subjects satisfied the clinical criteria for asthma and had the relevant inflammatory process. Of course, the study is not of the entire asthmatic population as some individuals will remain undiagnosed while others will have been managed entirely in general practice, but all those referred to hospital and willing were entered into the study. We believe that the referral threshold was relatively low, and very few patients were referred outwith the local area. The subjects were recruited by a single handed hospital physician, with no other selection. There were no differences in the demography or function of those entering at different times [11]. Most were stable on maintenance corticosteroids [8].

The rate of decline of actual FEV1 was similar to that reported by Ulrik [1] (38 ml/year) and Lange [2] (50 ml/year). This study demonstrates for the first time that decline in best after bronchodilator function, the definitive physiological measurement for COPD [16], is similar to that of actual function. Actual/best PEF tended to improve over the period [8], so measurement of actual FEV1 potentially under-estimates any decline in best FEV1, but in practice loss of actual FEV1was not relevantly different from that of best FEV1.

As in the previous studies [1,2] the rate of decline of actual FEV1 is greater than that suggested by reference equations [12,17] which are derived from cross-sectional data. Values derived from longitudinal data may differ from cross-sectional observations for a number of reasons [18,19]. These include a cohort effect and, with lung function, loss of height with ageing which will mask decline in the cross sectional tables. We allowed for loss of height by using height at the start of each 5-year period. After discounting this there was little difference between the genders, the principal association with change in function being the age of the subjects. Longitudinal studies suggest increased loss of FEV1 in elderly normal subjects [20-22], but the rate of decline in our study is greater at all ages than that reported from the general population [20]. Our results suggest that that a cubic model involving age is appropriate for all of the three measures of respiratory function that we have analysed. Although the pattern in a particular individual cannot be determined from these computed curves, they strongly suggest that decline is not linear. The interpretation of the pattern of change is critically dependent on its normal course. The critical points on the curve include the age at which maximum life-time function is achieved and the possibility of a plateau, before deterioration starts The age when personal best is achieved is variously estimated between the early twenties and the late thirties [19], and may well vary from person to person. In looking for a plateau phase Robbins et al [23] demonstrated both positive and negative slopes in different individuals. Any decline seen where improvement or no change is anticipated must be excessive and so the slow decline that we observed in the third decade may be an under-representation of the true loss of function. It is more difficult to explain the faster decline in early compared to late middle age. As the entry criteria of this study were a diagnosis of asthma with no attempt to exclude co-existent COPD, it is possible that this reflects a period of life when the effects of the inflammatory process associated with COPD are particularly apparent. In subjects where asthma and COPD co-exist, COPD might contribute to presentation in these subjects, and decline in function might be particularly rapid especially if there were interaction between the two inflammatory processes. The above is compatible with the apparently unsustainable rates of decline observed early in COPD as in the Euroscop study [24]. We confirmed that decline accelerates after in late life in these asthmatics as it does in normal subjects [[20,21], and [22]].

Outcome in successive periods is necessarily confounded by the effects of management and attrition. Attrition is particularly relevant to the analysis of change in function, as the most powerful predictor of survival in these subjects is best function [11]. Higher dose of inhaled corticosteroids at the start of an observation period was not associated with better outcome within the same period. This is unsurprising because a higher dose reflects more severe disease. More surprisingly, there was also no evidence that the higher average dose in successive periods was associated with a favourable secular trend in observed, or maximum, function. Nevertheless in contrast, actual/best peak flow improved [8] and standardised mortality declined with time [11]. The latter was reduced twofold although subjects on higher therapeutic steps remained more likely to die even after allowance for other risk factors including best function. It may be that clinicians are able to recognise subjects of poor prognosis irrespective of function, but are more successful in reducing mortality than loss of function by adjustment of therapy. None of these considerations necessarily imply that the use of inhaled corticosteroids is ineffective in terms of function. As there were, of necessity, no controls, any effect of treatment, dictated by clinical need, is impossible to confirm. Improvement in functional outcome might have been confounded by reduced mortality in those with severe disease, or the dose response curves for reduction of mortality might be very different from those for prevention of airway remodelling.

We depended primarily on patient recall in making a diagnosis of childhood asthma. Although we had a low threshold for accepting the diagnosis, presumably recall would be more consistent when childhood symptoms were severe. This might produce a bias in favour of demonstrating associations. Nevertheless the univariate associations with change in function were stronger in those who claimed that they had had persistent symptoms since childhood, than in those who recalled childhood asthma after a gap of at least five years. The outcome in gap asthmatics was similar to those with adult onset, but surprisingly subjects with symptoms persistent since childhood showed a more favourable trend. This appears to be inconsistent with the long established observation that childhood asthma compromises adolescent and early adult lung function and that this is related to persistent symptoms [25,26]. However duration is probably the critical factor. The history of asthma is likely to be long in adults with symptoms persistent from childhood and rapid decline is shown to be associated with short duration. Although these subjects did have relatively poor lung function at entry to the study [10], probably reflecting their function on reaching maturity, it does not necessarily follow that retarded development will be succeeded by an excessive rate of decline later in life. It may be that at any age whether in childhood or adulthood the first decade of the disease is critical in determining loss of function. This might not be true in those with pure COPD, and explain why our findings are contrary to the 'horseracing effect' (the horse that runs fastest continues to extend its lead) as described by Fletcher in his classical population studies [27]. There the comparison was with normal subjects rather than those with established airway disease with differing lengths of history.

The effect of smoking appeared small, but there were few current smokers and the tobacco load was light. The study was not designed to observe the effects of tobacco smoking in asthma; the separate analysis of non-smokers was intended to describe the decline in the function of asthmatics unencumbered by the effects of cigarette smoking. It is inevitable that we have underestimated the potential association between smoking and decline in function in asthma and so do not suggest that the effect of smoking in asthmatics in general is unimportant.

The association between low initial actual/best function, implying poor control, and apparently favourable outcome is highly likely to reflect response to treatment. There was a strong relationship between low social class and poor control in the 1983 entry [10]. This may account for some of the paradoxical benefit of lower social class, possibly even hiding a real disadvantage.

## Conclusions

We present the decline in function in a dynamic cohort of adult asthmatics observed over fifteen years. The majority were treated with inhaled corticosteroids throughout the period. As there are no internal asthmatic or normal controls our study cannot determine definitively whether there is excess decline in the pulmonary function of adults managed conventionally with inhaled cortico-steroids. It does suggest, however, that the dose of inhaled steroids may not be critical over the recommended range. Our study confirms that the pattern of decline in actual and best ventilatory function is similar. This is important when comparing this study with epidemiological exercises where actual rather than best function has been measured. Furthermore these original findings in respect of the cubic effect of age should be taken into account when interpreting other articles reporting age effects on function, particularly where analysis of cross-sectional observations may imply that decline in the function is linear.

## Competing interests

The author(s) declare that they have no competing interests.

## Authors' contributions

CKC conceived the study and was responsible for design of data forms, recruitment and all the clinical aspects. RJP undertook the analysis of data. Both authors read and approved the final manuscript.

## Pre-publication history

The pre-publication history for this paper can be accessed here:


